# Articles of Significant Interest in This Issue

**DOI:** 10.1128/iai.00058-25

**Published:** 2025-02-18

## *CORYNEBACTERIUM* INHABITING THE UPPER AIRWAY KEEP BACTERIAL PATHOGENS IN CHECK

Greater abundance of airway *Corynebacterium* is associated with lower pneumonia risk. However, little is known regarding how *Corynebacterium* influence pneumonia development. Tamkin et al. (e00445-24) discovered a novel *Corynebacterium*-secreted factor which blocked the activity of *Staphylococcus aureus* pore-forming toxins. In contrast, a *Corynebacterium-*secreted lipase-inhibited growth of *Streptococcus pneumoniae* without affecting *S. aureus*. Ultimately, the presence of *Corynebacterium* reduced the ability of both pathogens to adhere to the epithelium and infect the lungs. These observations highlight the antimicrobial and anti-virulence properties of *Corynebacterium* as potential mechanisms contributing to baseline defense against bacterial pneumonia by the airway microbiome.

## THE INTERPLAY BETWEEN *ACINETOBACTER BAUMANNII* ZIGA AND SLTB PROMOTES ZINC HOMEOSTASIS AND CELL ENVELOPE INTEGRITY

This study elucidates the mechanisms by which bacteria coordinate metal availability with cell envelope integrity during infection. In the opportunistic human pathogen *Acinetobacter baumannii*, the zinc-responsive protein ZigA engages in a functional interaction with SltB, a cell wall-modifying that preserves cellular stability during zinc limitation. Disruption of this interaction compromises cell envelope architecture and impairs bacterial survival during infection. Critchlow et al. (e00422-24) uncover a previously unrecognized link between metal homeostasis and cell envelope maintenance, offering critical insights into the adaptive strategies employed by bacterial pathogens to thrive in metal-restricted host environments.

